# Does the line-to-line cementing technique of the femoral stem create an adequate cement mantle?

**DOI:** 10.1177/1120700020934368

**Published:** 2020-06-18

**Authors:** Kirsti Sevaldsen, Otto S Husby, Øystein B Lian, Vigdis S Husby

**Affiliations:** 1 Department of Orthopaedic Surgery, Kristiansund Hospital, Kristiansund, Norway; 2 Department of Neuromedicine and Movement Science, Norwegian University of Science and Technology, NTNU, Trondheim, Norway; 3 Department of Orthopaedic Surgery, Clinic of Orthopaedics, Rheumatology and Dermatology, St. Olavs hospital HF, Trondheim, Norway; 4 Department of Circulation and Medical Imaging, Norwegian University of Science and Technology, NTNU, Trondheim, Norway; 5 Faculty of Health Sciences, OsloMet – Oslo Metropolitan University, Norway

**Keywords:** Bone cement, cementing technique, French paradox, hip arthroplasty

## Abstract

**Background:**

The line-to-line cementing technique is proposed to create a press-fit in the femoral canal, which is contrary to modern cementing techniques. The term ‘French paradox’ has been used to describe the acceptable results associated with this technique. It has been suggested that the quality of the mantle may not be satisfactory, predisposing to early failure and aseptic loosening.

**Methods:**

The line-to-line cementing technique, where the femoral stem was oversized by 1 size compared to the broach, was compared to the standard cementing technique using corresponding sized broaches and stems, in 6 pairs of human cadaver femora with taper-slip design C-stems. Cement pressure was measured, and cement mantle thickness was analysed. A mixed effects model with random intercepts was used to examine the relationship between thickness of mantle and cementing technique and between pressure and cementing technique.

**Results:**

Line-to-line cementing results in significantly higher pressurisation for longer periods of time leading to better interdigitation but a thinner mantle in some areas.

**Conclusions:**

The results of this study describe the in-vitro advantages and disadvantages of the line-to-line cementing technique.

## Introduction

Total hip arthroplasty (THA) is 1 of the most successful procedures in orthopaedic surgery with relatively low complication rates and a high degree of patient satisfaction.^1,2^ Very thin cement mantle thickness and defects in its substance have been correlated to possible early failure and aseptic loosening.^3,4^ The purpose of the line-to-line cementing technique (line-to-line) is to achieve an optimal micro interlock between cement and bone and to create a fitting composite mantle envelope.^5^ This surgical technique stands in prominent contradiction to the philosophy of current cementing technique resulting in a cement mantle thickness of 2–4 mm by using an smaller stem than broach.^6,7^

2 French designed cemented femoral stems, the Ceraver Osteal and Charnley-Kerboull (CK),^8,9^ were both designed to achieve direct load transfer from the stem to the femoral bone structure.^10^ This design philosophy results in a very thin cement mantle that is even incomplete in some Gruen zones.^11,12^ Excellent survival rates were reported for the polished and rectangular double tapered CK mark I and II.^13^

Several studies have investigated the line-to-line versus the standard cementing technique (standard), but there appears to be a need to explore the relationship between cement pressure and cement mantle thickness in the 2 cementing philosophies.

The aim of the study was to measure the differences in cement pressure and cement mantle thickness by comparing the line-to-line and the standard cementing of the femoral stem in hip arthroplasty.

The hypothesis is that there is no significant difference in cement pressure and cement mantle thickness between the line-to-line and the standard cementing.

## Methods

This experimental research study was designed as an in vitro controlled laboratory study with between-group comparisons. 12 cadaver femora (6 pairs) were prepared after x-ray templating for the polished C-stem acrylic copies (DePuy, Warsaw, Indiana, USA) with 9/10 taper, and resected 10 cm proximally to the medial femoral condyle and mounted in a jig. Femoral neck osteotomy was performed 15 mm above the trochanter minor. Following templating, both femora were prepared with the same broach. The 6 right femora received the corresponding implant, i.e., 1 mm undersized in all directions. The left femora received an implant with the same geometrical size as the broach, i.e., line-to-line.

**Figure 1.**
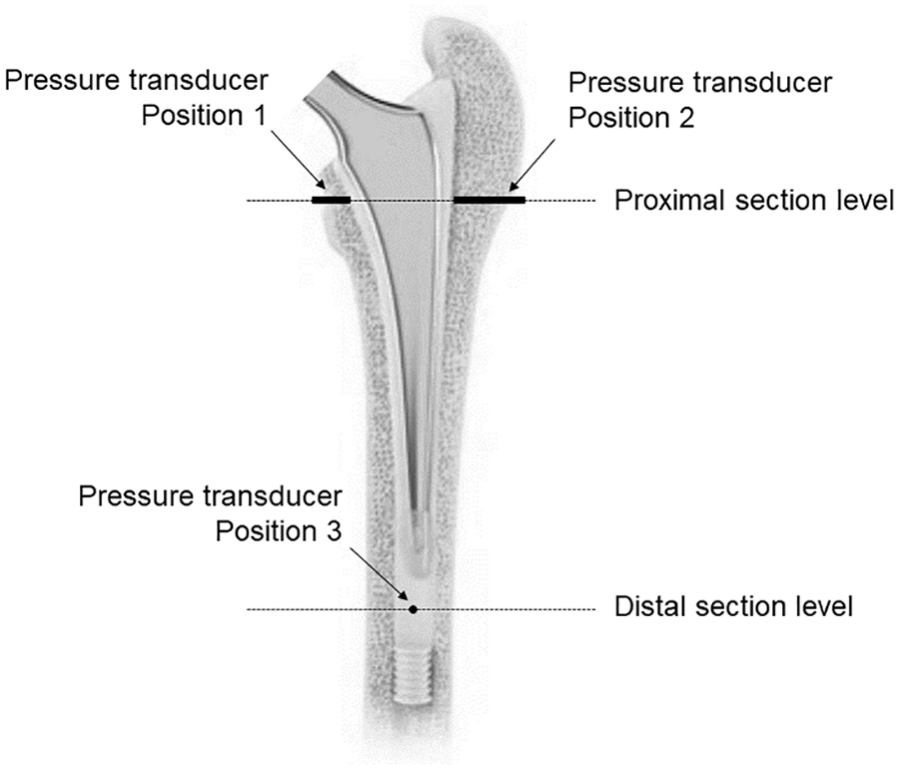
Cemented femoral stem with pressure transducers and section levels.

A distal polyethylene plug (DePuy, Warsaw, Indiana, USA) was inserted into the femoral canal to achieve a 15-mm distance between the tip and plug.^14^ To measure cement pressure, 3 pressure transducers (PWFD-PB, Tokyo Sokki Kenkyujo) were treaded into the specimens (Figure 1). The positions were standardised to 15 mm medial distal to the calcar (Position 1), 15 mm lateral distal to the trochanter major (Position 2) and 10 mm anterior proximal to the cement plug (Position 3).

40 grams of Smartset GHV (DePuy, Warsaw, Indiana, USA) cement stored at 18°C was mixed using the Cemvac mixing system (DePuy, Warsaw, Indiana, USA) under vacuum at 0.92 bar in a temperature controlled area at 19°C.^15^ The hardening time of the SmartSet GHV high viscose cement is 13.5 minutes at 19°C. At 150 seconds after the start of mixing the cement was filled retrograde into the femoral canal and pressurised using a Cemvac 1 cement gun with cement pressuriser and 15-mm nozzle. In the 6 right femora, the prosthesis was implanted by hand using the manufacturer’s stem introducer and in the 6 left femora by hammer strokes.

**Figure 2.**
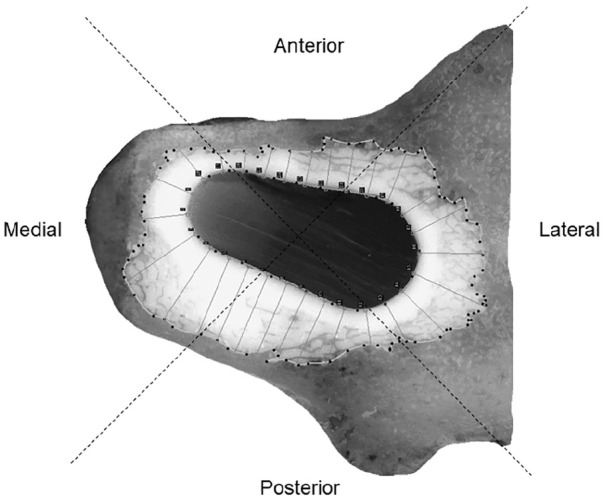
Transversal proximal section level indicating anatomical locations and spikes.

The pressure-time curves were recorded throughout the procedure of prosthesis implantation. A 4-Channel, 24-Bit Half/Full-Bridge Analog Input Device (NI USB-9237, National Instruments) was used for the pressure measurements. The femora were sectioned at the same level as the transducers (Figure 1). The cement mantle thickness was measured by drawing 28 perpendicular lines from the surface of the stem to the outmost visual border of the mantle (Figure 2).

### Risk assessments

The femoral specimens were anonymised. 3 male and 3 female pairs of femora aged between 50 and 70 years were used. Gender and age of the specimens are known to secure a proper bone quality as females above 75 years have higher degrees of osteoporosis than men at the same age.

The study was evaluated by the Regional Committees for Medical and Health Research Ethics in Norway, and an application was not needed (REC number: 2017/1409/ REC Central Norway).

### Statistical analysis

To examine the relationship between the thickness of the mantle and the cementing technique, a mixed effects model with random intercepts was estimated. Mantle thickness was the dependent variable, and cementing technique was the independent variable. In addition, anatomical locations in the sagittal and transversal planes were included as independent covariates. The model allowed for all possible interaction effects among the 3 independent variables.

To examine the relationship between pressure and cementing technique, another mixed effects model with random intercepts was estimated. Here, logarithmic pressure was the dependent variable, and the cementing technique and anatomical location in the transversal plane were independent variables. The anatomical location in the sagittal plane was not included in this model, as analyses showed that there were no signs of variations in pressure with this plane. The model allowed for possible interaction effects between the 2 independent variables.

Assuming a standard deviation of mantle thickness equal to 0.4 and a standard deviation of log-pressure equal to 0.2, the required sample size to document differences in mantle thickness and log pressure of 1 and 0.5, respectively, at a power of 80% and an adjusted significance level of 2.5% is 10 femora. Thus, the chosen sample of 12 femora is adequate.

Estimated marginal means were calculated from the fitted models, and pairwise comparisons were made using Bonferroni to adjust for multiple comparisons. All analyses were performed by using IBM SPSS Statistics for Windows, Version 25.0. Armonk, NY, US: IBM Corp.

## Results

The thicknesses of cement mantle varied significantly with cementing technique and anatomical location in the sagittal and transversal planes, respectively (Table 1). The line-to-line method yielded a statistically significantly thinner mantle compared to the standard for the proximal level for both posterior and lateral sides and the distal level on the medial side. For the distal level on the posterior and lateral sides and for the proximal level on the anterior and medial sides, the line-to-line resulted in statistically significant thicker mantle compared to the standard. There were no differences in thickness for the distal level on the posterior or the anterior side.

**Table 1.** Estimated marginal means of thickness of mantel.

Side	Level	Standard (CI)	Line-to-line (CI)	*p*-value^*^
Posterior	Proximal	4.98 (4.22–5.75)	3.83 (3.07–4.60)	<0.001
	Distal	5.45 (4.68–6.21)	5.16 (4.40–5.93)	0.313
Lateral	Proximal	6.48 (5.71–7.25)	4.75 (3.98–5.53)	<0.001
	Distal	2.65 (1.88–3.43)	3.87 (3.09–4.64)	<0.001
Anterior	Proximal	7.18 (6.41–7.94)	8.61 (7.84–9.37)	<0.001
	Distal	6.83 (6.06–7.59)	6.94 (6.18–7.71)	0.680
Medial	Proximal	6.92 (6.11–7.72)	7.46 (6.65–8.26)	<0.001
	Distal	8.75 (7.95–9.56)	6.55 (5.75–7.36)	<0.001

CI, confidence interval.

* *p*-value comparing difference in means adjusted for multiple comparisons using Bonferroni.

The logarithmic pressure varied significantly with the cementing technique and anatomical location in the transversal plane (Table 2). For all locations, the estimated pressure was higher for the line-to-line technique compared to the standard technique. The difference was statistically significant for the anterior location but was borderline significant for the lateral location. The difference was the smallest and non-significant for the medial side.

**Table 2.** Estimated marginal means of log pressure.

Side	Standard (CI)	Line-to-line (CI)	*p*-value^*^
Lateral	3.47 (3.16–3.79)	3.89 (3.58–4.20)	0.058
Anterior	4.24 (3.92–4.55)	4.82 (4.48–5.17)	0.013
Medial	3.64 (3.32–3.95)	3.91 (3.57–4.25)	0.223

CI, confidence interval.

* *p*-value comparing difference in means adjusted for multiple comparisons using Bonferroni.

**Figure 3.**
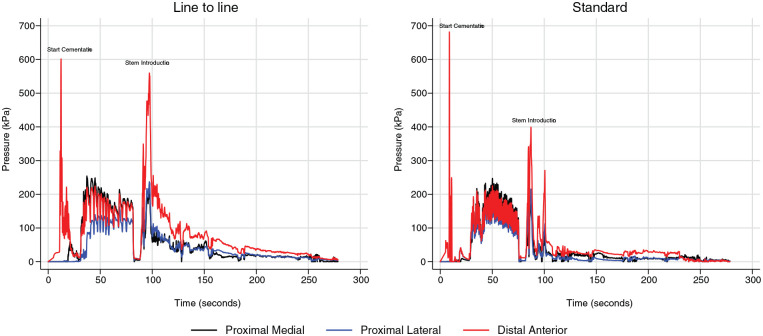
Typical pressure-time curves during cementing.

2 pressure peaks are noted during cement introduction and stem insertion (Figure 3). Compared to the standard, cement pressures are higher, and the pressure time durations are longer by using the line-to-line, during and after stem insertion. The mean peak pressure for stem insertion in the line-to-line was 187 kPa (standard deviation [SD] 42) for proximal medial, 189 kPa (SD 66) for proximal lateral and 468 kPa (SD 127) for lateral anterior. The corresponding mean peak pressure values for the standard were 179 kPa (SD 49) for proximal medial, 160 kPa (SD 54) for proximal lateral and 351 kPa (SD 96) for distal anterior.

## Discussion

To explore if the line-to-line method creates an adequate cement mantle, the line-to-line and the standard were compared in human cadaveric specimens by investigating the relationship between cementing technique and cement pressure and mantle thickness.

The main findings from this study were that the thickness of the cement mantle varied significantly with cementing technique and anatomical locations in the sagittal and transversal planes and that cement pressure varied significantly with cementing technique and anatomical location in the transversal plane.

It is reasonable to assume that cement penetration increases with increasing pressure. The mean peak pressure was significantly higher by the line-to-line compared to the standard. Therefore cement penetration is probably higher for the line-to-line. A previous study shows that the mantles were of similar thickness in the two techniques but interdigitated with the bone to a greater extent with the line-to-line.^16^

Broaching will remove and impact cancellous bone. The anatomical shape of the broach determines the cavity; therefore, cancellous bone remains proximal. The pressure is lower proximally than distally in both cementing techniques. Distally to the tip and upward, most of the cancellous bone will be removed, and the cement will move towards the cortical bone. One can assume that it is not possible to achieve similar high pressures proximally due to cement backflow and interdigitation. Around the tip of the prosthesis, the pressure is higher in both cementing techniques compared to the proximal pressures, but significantly higher for the line-to-line. This finding may be caused by an occlusion effect due to the prosthesis, the cement plug and the stem centraliser. The pressure is higher at the onset of cementing, pressurisation and introduction of the stem with the line-to-line. Additionally, the pressure after stem insertion is higher with the line-to-line, and remains longer at a higher level.

Using an oversized stem, higher pressure will be generated by the introduction of the stem itself by displacing a larger volume of cement.^17^

For the line-to-line, the cement will have a narrower outflow channel between prosthesis and bone compared to the standard. Thus, the cement must work against a higher resistance to flow back and subsequently increase the pressure. This may explain the lower rate of pressure reduction. Higher pressure forces the cement to penetrate to a greater extent, and the pressure remains longer at a higher level. One achieves a high degree of interdigitation and micro-interlock between cement and bone by using the line-to-line. The mantle becomes thinner in several areas but penetrates better. The cement-bone interface that encloses the prosthesis will act as a press-fit and might prevent micro-movements of the prosthesis. This finding is consistent with the findings of another study that observed that the insertion of the stem generated the highest pressure. The press-fit technique immobilised all the cement interfaces during cement hardening. It was suggested that eliminating micro-movement during this period has profound and long-lasting effects on long-term survival of the stem.^18^ Canal-filling stems supported by cortical bone have been reported to rotate less and create less cracks resulting in a superior cement mantle compared to trabecular bone-supported implants.^19^ There is also a geometrical factor; a larger stem has a greater surface to transfer forces to the composite mantle. This corresponds to the findings of a study that proposed that canal filling stems perform well due to the relatively low cement stresses and increased stability of the larger implants.^20^

The pressures are never as high with the standard and the time duration is shorter compared to the line-to-line. The pressure decreases at a faster rate after the stem introduction. This result may be because the cement flows back more efficiently. The envelope that encloses the prosthesis may become less optimal and may allow for more micro-movements of the stem. It has been shown in vivo that the extent of the pressurisation of cement into bone is the most crucial determinant of failure at the cement-bone interface.^21^

Bone cement implantation syndrome (BCIS) is a potentially life-threatening complication that may occur in cemented hip arthroplasty. Increased intramedullary pressure forces debris, bone marrow and fat into the systemic vascular circulation, and causes hypoxia, hypotension, cardiac arrhythmia, embolisation of the pulmonary vascular system and cardiac arrest. The degree of embolisation appears to be related to peak pressure in the femoral canal during pressurisation and cementation. With the line-to-line, the pressure is higher and lasts longer than with the standard. There is a risk of BCIS in both cementing techniques, presumably higher with the line-to-line.^22^ However, the use of pulsatile lavage decreases the risk of BCIS.^23^

In this study, a matched pair technique was used to limit the effect of specimen variability. X-ray templating served as a reference for size similarity of the paired specimens.

The use of human cadaveric bone includes cancellous bone for cement interdigitation, which is of importance to recreate a complex composite mantle. Cadaveric tissue has structural and material properties that resembles as much as possible clinical reality. Contrary to other studies, mantle thickness was investigated in relation to anatomical locations of the femur.

The study was limited by the fact that it was performed on relatively few human cadaver specimens. Cross-sections of the specimens were performed proximally and distally, and the mantle of other parts of the implant was not taken into consideration. The results and conclusions only apply to taper-shaped polished implants. Variations in implant design and surface roughness were not investigated.

Our data indicate that higher cement pressure leads to increased cement penetration possibly through cancellous bone to the internal surface of the cortex.

Future investigations on this topic may include a randomised controlled roentgen stereogrammatic analysis and clinical trial comparing femoral component migration between the line-to-line and the standard.

## Conclusion

In this study the method of implanting a prosthesis into a prepared cavity of a corresponding size generates higher cement pressure and increased penetration resulting in a thinner mantle with better interdigitation generally for the line-to-line compared to the standard. The results from this experiment indicate that the line-to-line is an adequate, if not superior, method for the implantation of taper-shaped polished femoral components in THA.
